# Encapsulation of Variabilin in Stearic Acid Solid Lipid Nanoparticles Enhances Its Anticancer Activity in Vitro

**DOI:** 10.3390/molecules25040830

**Published:** 2020-02-14

**Authors:** Mookho S. Lerata, Sarah D’Souza, Nicole R.S. Sibuyi, Admire Dube, Mervin Meyer, Toufiek Samaai, Edith M. Antunes, Denzil R. Beukes

**Affiliations:** 1 School of Pharmacy, University of the Western Cape, Bellville 7535, South Africa; 3520938@myuwc.ac.za (M.S.L.); adube@uwc.ac.za (A.D.); 2 Department of Science and Technology/Mintek Nanotechnology Innovation Centre (DST/Mintek NIC), Biolabels Node, Department of Biotechnology, University of the Western Cape, Bellville 7535, South Africamemeyer@uwc.ac.za (M.M.); 3 Department of Environmental Affairs, Oceans and Coasts Research Chief Directorate, Marine Biodiversity and Ecosystems Research, Victoria & Alfred Waterfront, P.O. Box 52126, Cape Town 8000, South Africa; tsamaai@environment.gov.za; 4 Department of Biodiversity and Conservation Biology, University of the Western Cape, Bellville 7535, South Africa; 5 Department of Biological Sciences, University of Cape Town, Private Bag X3, Rondebosch, Cape Town 7701, South Africa; 6 Department of Chemistry, University of the Western Cape, Bellville 7535, South Africa; ebeukes@uwc.ac.za

**Keywords:** marine natural products, variabilin, *Ircinia* sp., sponge, solid lipid nanoparticles, anti-cancer activity

## Abstract

The use of natural products as chemotherapeutic agents is well established; however, many of these are associated with undesirable side effects, including high toxicity and instability. Furthermore, the development of drug resistant cancers makes the search for new anticancer lead compounds a priority. In this study, the extraction of an *Ircinia* sp. sponge resulted in the isolation of an inseparable mixture of (7*E*,12*E*,20*Z*)-variabilin (**1**) and (7*E*,12*Z*,20*Z*)-variabilin (**2**) and structural assignment was established using standard 1D and 2D NMR experiments. The cytotoxic activity of the compound against three solid tumour cell lines displayed moderate anti-cancer activity through apoptosis, together with a general lack of selectivity among the cancer cell lines studied. Structural assignment and cytotoxic evaluation of variabilin was complicated and further aggravated by its inherent instability. Variabilin was therefore incorporated into solid lipid nanoparticles (SLNs) and the stability and cytotoxic activity evaluated. Encapsulation of variabilin into SLNs led to a marked improvement in stability of the natural product coupled with enhanced cytotoxic activity, particularly against the prostate (PC-3) cancer cell line, with IC_50_ values of 87.74 μM vs. 8.94 μM for the variabilin alone and Var-SLN, respectively. Both variabilin and Var-SLN revealed comparable activity to Ceramide against the MCF-7 breast cancer cell line, revealing IC_50_ values of 34.8, 38.1 and 33.6 μM for variabilin, Var-SLN and Ceramide, respectively. These samples revealed no activity (>100 μM for all) against HT-29 (colon) cell lines and MCF-12 (normal breast) cell lines. Var-SLNs induced 47, 48 and 59% of apoptosis in HT-29, MCF-7 and PC-3 cells, respectively, while variabilin alone revealed 38, 29 and 29% apoptotic cells for HT-29, MCF-7 and PC-3 cell lines, respectively. The encapsulation of natural products into SLNs may provide a promising approach to overcome some of the issues hindering the development of new anticancer drugs from natural products.

## 1. Introduction

Cancer continues to be one of the leading causes of death and is a major health burden worldwide [1]. Breast cancer, the most diagnosed cancer [2], accounts for 25% of all diagnosed cancers with 20% malignancy related mortalities in African women [3], while prostate cancer has the highest incidence rate reported among African men [4]. The burden of cancer is only expected to increase among low- and middle-income populations due to adaptation of western lifestyles and increased life expectancy [5]. Given the increasing prevalence of cancer and the demonstrated relevance of natural products as a source of anticancer drugs, we embarked on a program to explore the anticancer potential of South African marine sponge natural products against breast (MCF-7), colon (HT-29) and prostate (PC-3) cancer cell lines.

**Figure 1:**
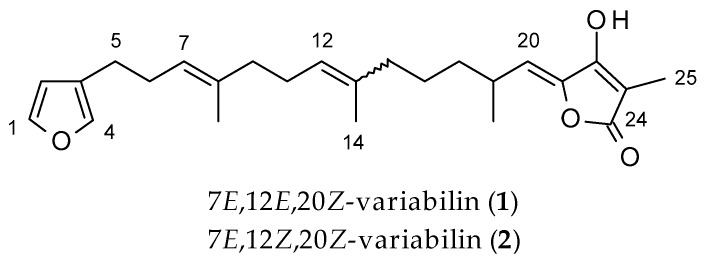
Structure of a mixture of 7*E*,12*E*,20*Z*-variabilin (**1**) and 7*E*,12*Z*,20*Z*-variabilin (**2**).

Furanosesterterpenes, such as 7*E*,12*E*,20*Z*,18*S*-variabilin (**1**) (Figure 1) and its isomers have been shown to exhibit cytotoxic activities against several human cancer cell lines for example, lung (A549), ovarian (SK-OV-3), skin (SK-MEL-2), central nervous system (CNS, XF498) and colon (HCT15) cancer cell lines [6,7,8]. However, furanosesterterpenes such as **1**, bearing tetronic acid and furan moieties, are unstable and decompose readily in the presence of light and air, resulting in decreased cytotoxic activity [9]. One strategy that may be used to increase the stability of unstable natural products and obtain more accurate biological activity data, is to incorporate them into nanoparticles.

SLNs are nanometer sized particulate drug delivery systems, typically ranging in size from 50–100 nm. Such nanoparticle systems are an advancement over conventional drug delivery systems such as emulsions and liposomes [10]. SLNs consist of spherical bodies with a solid lipid core (within which the drug is located depending on its hydrophobicity or hydrophilicity) surrounded by a surfactant [11]. These particles offer several advantages including providing protection to compounds sensitive to unfavorable environmental factors such as moisture, light and pH which would result in degradation and a significant loss in biological activity [12]. Additionally, SLNs have also improved the biocompatibility and delivery of poorly water-soluble anticancer drugs such as paclitaxel and camptothecin [13].

The main aim of the current study was to identify the main cytotoxic component in the organic extract of the South African marine sponge, *Ircinia* sp. (Figure S1) and to improve on the main metabolite’s stability and cytotoxicity by incorporation into solid lipid nanoparticles.

## 2. Results and Discussion

### 2.1. Isolation and Characterization of 7E,12E,20Z- and 7E,12Z,20Z-Variabilin (***1*** and ***2***)

The *Ircinia* sponge (collection code: TS2713) was selected for study on the basis of its preliminary cytotoxic activity (>50% growth inhibition at 50 μg/mL) against an MCF-7 breast cancer cell line. The crude organic extract was initially fractionated on a Diaion HP20 resin followed by further purification using silica gel column chromatography and normal phase HPLC to give a fraction containing **1** and **2** as the major compounds. Further attempts to separate compounds **1** and **2** were unsuccessful and led to their degradation. The compounds were thus analysed as a mixture. High resolution electrospray ionization mass spectrometry (HR-ESIMS) revealed a protonated molecular ion at *m*/*z* 399.2532 (calcd. 399.2537), corresponding to a molecular formula of C_25_H_34_O_4_, for isomers (**1** and **2**). The ^1^H NMR spectrum (Figure S2) showed a number of overlapping signals of two very similar compounds. Careful analysis of 1D and 2D NMR spectra revealed the sample to be a 1:3 mixture of 7*E*,12*E*,20*Z*- and 7*E*,12*Z*,20*Z*-variabilin (**1** and **2**, respectively). These compounds were previously isolated by Höller et al. as their methyl ether derivatives where, following fractionation by normal phase VLC, diazomethane was used to methylate the tetronic acid fraction. The fraction was then subjected to further fractionation and finally normal phase HPLC [14].

### 2.2. Synthesis and Characterization of Var-SLNs

**Figure 2:**
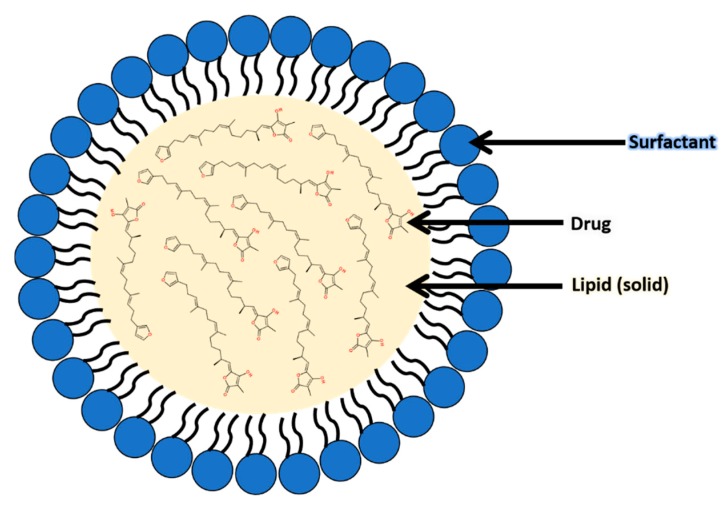
Schematic of a variabilin-loaded solid lipid nanoparticle.

The poor stability of compounds **1** and **2** suggested that its cytotoxic activity is possibly underestimated. Therefore, in order to improve the stability of **1** and **2** and obtain more accurate biological data, we encapsulated the mixture into stearic acid/poloxomer 188 SLNs (Var-SLNs) using a hot homogenization method. Figure 2 shows a schematic representation of the natural product incorporated into the SLN.

**Figure 3:**
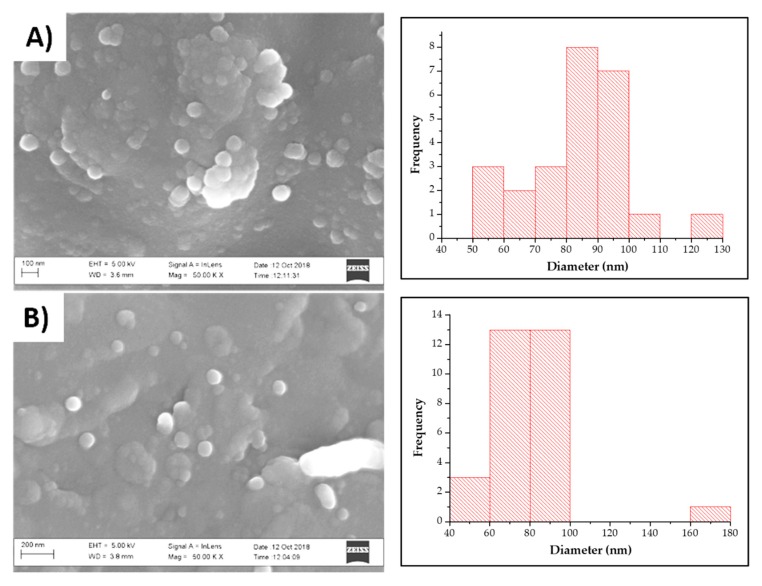
SEM images of Variabilin Loaded Stearic Acid Solid Lipid Nanoparticles (Var-SLNs) (**A**) and the blank SLNs (**B**) together with their corresponding diameter histograms.

Scanning electron microscopy revealed the nanoparticles to have a smooth, spherical surface, with an average size of 83.5 nm and 81.8 nm in size (Figure 3) for the Var-SLNs (Figure 3A) and the SLNs prepared without variabilin (Figure 3B), respectively. The associated histograms for the SLNs show a broader size distribution for the SLNs prepared without variabilin.

The polydispersity index (PDI), hydrodynamic radii and the zeta potential of the SLNs were measured (Table 1) at room temperature using a Zetasizer. The samples produced a homogenous, milky suspension when dispersed in water. Smaller sized particles were obtained for the Var-SLNs with hydrodynamic radii at 198.2 nm, while the Blank-SLNs revealed hydrodynamic radii of 322.8 nm (Table 1). The hydrodynamic radii were measured again 2 weeks later and increases were observed for both the Var-SLNs and the blank sample, with the latter showing a pronounced increase of over 500 nm (Table 1). Zeta potential measurements demonstrated that the surface of the nanoparticles is negatively charged at -31.4 mV and -18.3 mV for the Var-SLNs and the Blank-SLNs, respectively, as expected since stearic acid (pK_a_ 4.75) in a solution at pH 7.4 is negatively charged. This data also showed that the Var-SLNs are more stable than the blank SLNs, since nanoparticles with zeta potential values greater than +30 mV, or less than -30 mV, correlates with inherent stability [15]. At the time of synthesizing the SLNs, the Var-SLNs and the Blank-SLNs showed PDI values (Table 1) of 0.129 and 0.161, respectively, which indicates that they have a narrow size distribution [16]. Measurement of these radii 2 weeks later (Table 1) revealed a slight increase for the Var-SLNs (at 0.136, Table 1), where the Var-SLNs retained their narrow size distribution, while the blank-SLNs showed a significant change at 0.336 (Table 1). The hydrodynamic radii of the particles are larger than 100 nm, and the samples may therefore fall in the meso- rather than nano-scale, as is often observed with colloidal suspensions.

**Figure 4:**
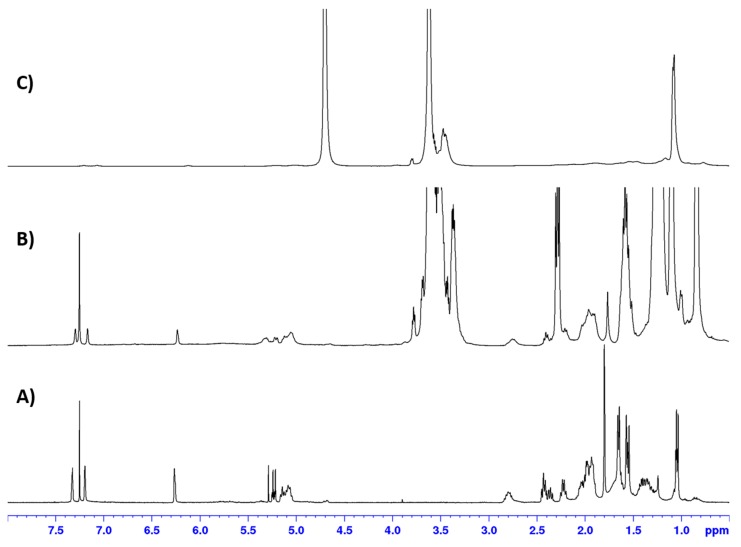
^1^H NMR spectra (CDCl_3_, 400 MHz) for mixture of **1** and **2** (**A**), Var-SLNs, (**B**), and the poloxamer 188 copolymer (**C**).

Successful inclusion of the mixture of **1** and **2** into the SLNs (Var-SLNs) was confirmed through extraction of the nanoparticles with chloroform and analysing this extract using ^1^H NMR spectroscopy (Figure 4). The ^1^H NMR spectra revealed signals attributed to the furan moiety of variabilin, amongst others, confirming the inclusion of variabilin into the SLNs (Figure 4B).

**Figure 5:**
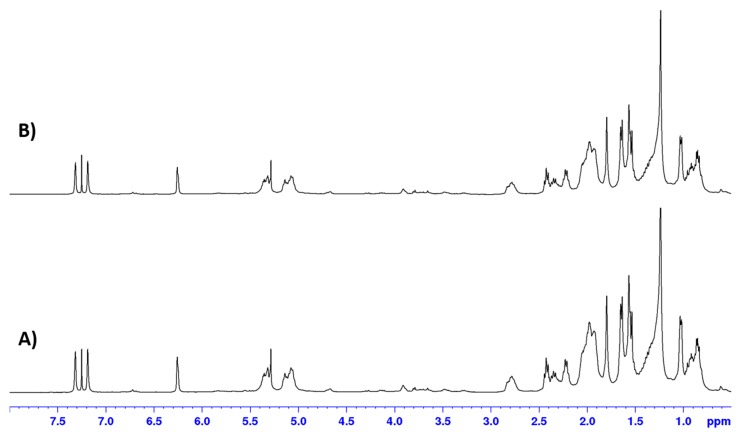
^1^H NMR spectra (CDCl_3_, 400 MHz) for the Var-SLNs immediately after purification (**A**), and one week later (**B**).

The ^1^H NMR spectra also revealed a remarkable improvement in the stability of variabilin following incorporation into SLNs. Figure 5 shows the ^1^H NMR spectra obtained for variabilin incorporated into a solid lipid vehicle immediately after preparation (Figure 5A), while a similar ^1^H NMR spectrum was obtained from the same Var-SLNs, one week later (Figure 5B).

### 2.3. Cytotoxic Activity of the Mixture of ***1*** and ***2*** and Var-SLNs

**Figure 6:**
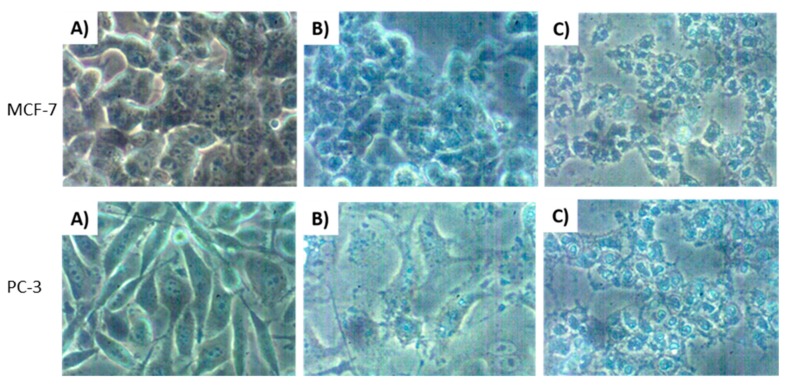
Morphological changes in MCF-7 and PC-3 cells 24 h after treatment. Cells in (**A**) were left untreated, while cells in (**B**) represent the cells treated with 60 μM ceramide and (**C**) cells treated with 100 μM of variabilin. Cellular morphology was observed and captured at 20× magnification using an inverted (Nikon) light microscope.

**Figure 7:**
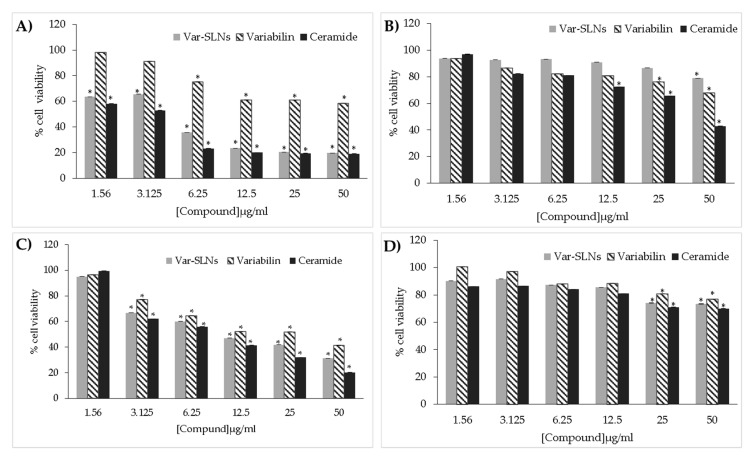
Effect of variabilin and Var-SLNs against cancer and non-cancer cell lines. The cells: PC-3 cells (**A**), MCF-12 (**B**), MCF-7 (**C**), and HT-29 (**D**) were exposed to increasing concentrations of variabilin and Var-SLNs, cell viability was assessed by WST-1 assay. * Statistical significance (*p* < 0.05).

Breast (MCF-7), colon (HT-29) and prostate (PC-3) cancer cell lines, as well as normal breast cell lines were cultured to approximately 90% confluence prior to treatment with increasing concentrations of variabilin (**1**, **2**) and Var-SLNs. The cytotoxicity of the compounds was determined using a water soluble tetrazolium salt (WST-1) proliferation assay, while the apoptotic assays were carried out using an APOPercentageTM dye. Figure 6 shows typical morphological changes induced by 100 µM of variabilin, similar effects as observed in MCF-7 and PC-3 cells (Figure 6C) were seen in all the tested cells after 24 h of treatment. Ceramide was used as a positive control, its effects were more pronounced on PC-3 cells at 60 μM. As shown in Figure 7, variabilin and Var-SLNs induced a dose response against the treated cells.

The cytotoxic activity of Var-SLNs was evaluated and compared to the natural product alone. In general, incorporation of variabilin into the lipid nanoparticle resulted in a decrease in cell viability in all three cancer cell lines (Figure 7). The most pronounced effect was seen in the prostate cancer cells (PC-3) where the Var-SLN was almost ten times more cytotoxic than the natural product alone, as clarified by the IC_50_ values listed in Table 2. Interestingly, the Var-SLN appears to have a protective effect on the non-cancerous MCF-12A cell line and increased the viability of the cells. The SLN without the natural product had no effect on cell viability at the concentrations tested.

### 2.4. Apoptotic Effects of Variabilin and Var-SLNs

Following the cytotoxic evaluation for variabilin and Var-SLNs against MCF-7, MCF-12A, PC-3 and HT-29 cells, it was evident that the compounds possessed some cytotoxic activity; therefore, the mechanism of cell death induced by variabilin was evaluated using APOPercentageTM dye. All cells were treated for 24 h at concentrations equivalent to their IC_50_ and 100 µM where IC_50_ exceeded 100 µM (Table 2). Positive control cells were treated with ceramide, while negative control cells were left untreated. To determine induction of apoptosis, cells were stained with APOPercentageTM dye and dye uptake was quantified using flow cytometry. Cells undergoing apoptosis possess certain characteristics such as shrinking and condensation of the nuclear chromatin, as well as lyses of nucleus [17,18].

**Figure 8:**
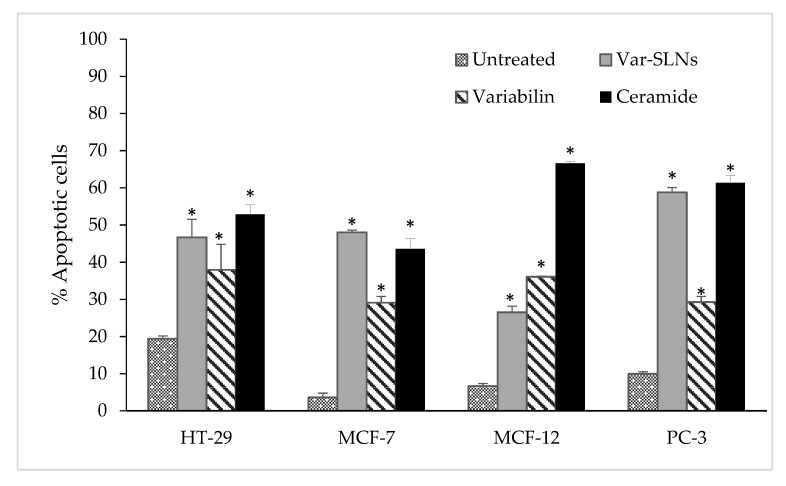
APOPercentageTM results obtained for variabilin (1) and Var-SLNs, together with ceramide (positive control) and untreated cells which served as a negative control. * Statistical significance (*p* < 0.05).

It was evident from the morphological changes observed for both MCF-7 and PC-3 cell lines, that variabilin induced apoptosis (Figure 6C). The compound and SLNs showed the least amount of apoptotic cells on MCF-7 cells, while PC-3 cells showed higher response to Var-SLNs. Similar to the cytotoxicity results Var-SLNs showed increased apoptosis in cancer cells when compared to non-cancer cells. Var-SLNs had an improved apoptotic activity on HT-29, MCF-7 and PC-3 cells, comparable to that of the positive control (ceramide). Var-SLNs induced 47, 48 and 59% of apoptosis in HT-29, MCF-7 and PC-3 cells, respectively; which was slightly better than ceramide on MCF-7 cells with 43% of apoptotic cells (Figure 8). The results also show that Var-SLNs induced a more significant apoptotic effects on PC-3 cells compared to other cancer cells, and selective apoptotic effects on the non-cancerous cells.

## 3. Materials and Methods

### 3.1. General Experimental Procedures

Column chromatography (SPE) of the sponge extract was performed on Supelco Dianion^®^ HP20SS (Bellefonte, PA, USA) and silica gel 60 (0.040–0.063 mm) from Merck KGaA (Darmstadt, Germany). Normal phase TLC was performed on Silica gel 60 F_254_ aluminium sheets purchased from Merck KGaA (Germany) and visualized under UV light at 254 and 365 nm. All high-performance liquid chromatography procedures were carried out with Agilent technologies equipped with ultraviolet and refractive index detectors using a Whatman 10 μm silica (2) semi-preparative column 50 cm × 10 mm (i.d.). NMR samples were prepared in deuterated solvents and all experiments acquired on a Bruker Avance III HD 400 MHz spectrometer equipped with a 5 mm BBO probe at 298K. Chemical shifts were referenced to deuterated solvent peaks (CDCl_3_ δ_H_ 7.25, δ_C_ 77.00) and reported in ppm. Homogenisation and sonication of the SLNs was accomplished using an IKA^®^ T18 digital Ultra Turrax^®^ homogenizer and Bandelin Sonoplus HD 2070, respectively. Cell studies were performed under a class II biological safety cabinet. To visualize the cells, a Nikon light microscope with 20x magnification was used together with a Leica EC digital camera.

### 3.2. Sponge Collection and Taxonomy

The sponge *Ircinia* sp. (specimen TS2713) was collected from a depth of 17 m from Grootbank, Plettenberg Bay (34°00.413′ S, 23°23.053′ E), South Africa by SCUBA in 2015. In life the sponge is massive and amorphous. The surface is rough with conulose ridges, covered with a sandy layer; and in situ the texture is very tough, firm, elastic, and extremely hard to tear or cut even when dry and in preservative. The specimen has medium to very coarse sand embedded in the base and sparsely distributed in the choanosome. The colour in life dark grey to charcoal black externally, internally golden-brown; in ethanol black/grey or brown. The preserved voucher sample is housed at DEA Oceans and Coast in the collection of Toufiek Samaai (TS). A subsample of specimen was stored at −20 °C until processed and a voucher specimen is kept in the Marine Biodiscovery laboratory at University of the Western Cape. The sponge is an undescribed species of the Family Irciniidae and was identified by Toufiek Samaai. The sponge used in this study belongs to the same genus and family of the species described by Su et al. (2011) [19].

### 3.3. Isolation and Characterization of Variabilin (***1*** and ***2***)

A frozen specimen of *Ircinia* sp. was thawed at room temperature prior to exhaustive extraction with 400 mL of CH_3_OH, followed by CH_3_OH:CH_2_Cl_2_ (1:1). Each solvent allowed the extraction of metabolites overnight, at room temperature, away from direct light. The extracts were combined and dried under reduced pressure and the resultant extract was subjected to a solid phase extraction with Dianion HP20ss using a stepwise gradient elution with CH_3_OH-H_2_O (0:100, 10:90, 20:80, 60:40, 20:80, 100:0), CH_3_OH-EtOAc (50:50) and EtOAc 100%. Thin layer chromatography (TLC) was performed on all 9 fractions. CH_3_OH-EtOAc 50:50 fraction was further separated on silica gel 60 (0.040–0.063 mm) and eluted with hexane-EtOAc (7:3) resulting in collection of 18 fractions which were later grouped, based on their TLC similarities, resulting in 7 fractions: 8A (1-2), 8B (3-4), 8C (5-8), 8D (9-10), 8E (11-14), 8F (15-17) and 8G (EtOAc wash). Fraction 8B was subjected to HPLC, using hexane-EtOAc (7:3) as the mobile phase at a flow rate of 3 mL/min. Variabilin (**1**) was collected as a mixture of geometric isomers at 14.50 min.

### 3.4. Preparation and Characterization of Variabilin Loaded Stearic Acid Solid Lipid Nanoparticles (Var-SLNs)

Var-SLNs were synthesized using a hot homogenization method adapted from the method described by Eskiler et al. [20]. Stearic acid (50 mg) was heated and melted at 75 °C and variabilin (25 mg), dissolved in dichloromethane (1 mL) added whilst stirring to form a stearic acid-variabilin complex with a mass ratio of 2:1. Separately, a poloxamer 188 (25 mg) solution in water was heated to 75 °C in water to achieve a final poloxamer 188 content dispersion of 5% *w*/*v*. This poloxamer solution was subsequently added to the stearic acid-variabilin mixture with constant stirring at 1000 rpm, maintaining the temperature at 80 °C for 5 min. The resultant mixture was then homogenized at 10,000 rpm for five minutes, followed by one minute of sonication and rapid cooling in an ice bath. Blank-SLNs were prepared using the same method but without any natural product. Var-SLNs and Blank-SLNs were prepared in a phosphate buffer solution (pH 7.4) and the size and polydispersity (PDI) and zeta potential of the SLNs were determined using a Malvern Zetasizer Nano ZS. High resolution scanning electron microscopy (LEO 1450 SEM, Zeiss, Oberkochen, Germany) images of the surface morphology of the nanoparticles were obtained using a Zeiss Gemini Auriga Scanning Electron Microscope equipped with a CDU-lead detector at 25 kV with tungsten filament. The freeze-dried Var-SLN and B-SLN samples were thinly spread on a slide, coated with carbon using argon and visualized using SEM. Encapsulation of variabilin was confirmed by extracting the Var-SLN with EtOAc. The extract was dried under reduced pressure and analysed using ^1^H NMR. Due to the small amounts of natural product available and the poor water solubility and stability of variabilin, drug release and encapsulation efficiency was not determined. Instead, 100% incorporation of variablin into the SLN was assumed for the calculation of variablin concentrations for cytotoxicity studies.

### 3.5. Cell Culture and Cytotoxicity Studies

Antiproliferation assays were done using a WST-1 (Roche, Grenzach, Germany) according to the method described by Mmola et al. with modifications [21]. MCF-7, MCF-12A, PC-3 and HT-29 cells were cultured in 96-well plates at a density of 1 × 10^5^ cells/mL and incubated in a humidified CO_2_ incubator at 37 °C for 24 h. Thereafter, the cells were treated with different concentrations (1.56–50 µg/mL) of variabilin and Var-SLNs, prepared by serial dilutions from stock solutions of 10mg/mL, also for 24 h. Positive control data was obtained by treating the cells with ceramide 0–80 µM, while the growth medium was used as a negative control. Following 24 h of treatment, the WST-1 dye was added to the cells and the cells incubated for 3 h. The absorbance of the dye was recorded at 440 nm, using 630 nm as a control, with an POLARstar Omega microplate reader (BMG Labtech, Ortenberg, Germany).

### 3.6. Apoptosis Assays

Apoptosis assay was performed using an APOPercentageTM dye which stains cells undergoing apoptosis according to the protocol described by Sibuyi et al. [22]. Cells were seeded in 12-well plates at a density of 2 × 10^5^ cells/mL and incubated for 24 h in a humidified CO_2_ incubator at 37 °C, after which the culture media was replaced with fresh media containing respective treatments (variabilin, Var-SLNs and ceramide) at a concentration equivalent to their IC_50_ and incubated for another 24 h. At the end of 24 h, culture media containing floating cells from each well were transferred to labelled centrifuge tubes, while adherent cells were obtained following gentle trypsinization and added to the corresponding centrifuge tubes. Cells were recovered by centrifugation, the supernatant was gently removed, the pellet resuspended in 250 µL of APOPercentageTM dye (diluted 1:160 in culture medium) and the cells incubated at 37 °C for 30 min. After incubation, 4 mL of PBS was added to each tube and the tubes were centrifuged for another 5 min at 3000 rpm. The supernatant was discarded, the pellet was resuspended in 300 µL of PBS and analysed for apoptosis using an Accuri C6 Plus flow cytometer (BD Sciences, San Jose, CA, USA).

### 3.7. Statistical Analyses

Student’s *t*-test and ANOVA was used (SPSS 7.5 software, Chicago, IL). Data are expressed as mean ± SD. Differences were considered statistically significant at a *p*-value ≤ 0.05.

## 4. Conclusions

Marine sponges are useful sources of potential anticancer drugs. However, some anticancer natural products suffer from poor solubility (or bioavailability) and stability problems. 12*E*- and 12*Z*-variabilin (**1** and **2**), successfully isolated from a sponge *Ircinia* sp. and characterized using standard NMR and mass spectroscopic techniques, revealed moderate anti-cancer activity against a three solid tumour cell lines (Prostate (PC-3), breast (MCF-7) and colon (HT-29)) as well as a normal breast (MCF-12) cell line. The inherent instability of compounds **1** and **2**, however, provided some cause for concern, since any bioactivity data obtained with the samples would be influenced by their instability. Encapsulation of variabilin into SLNs successfully improved the compound’s stability, with the Var-SLN sample showing a marked improvement in biological activity against the PC-3 and MCF-7 cell lines in particular. IC_50_ values of 87.74 μM vs. 8.94 μM against the PC-3 cell line were obtained for the variabilin alone and Var-SLN, respectively. The samples showed comparable activity to Ceramide against the MCF-7 breast cancer cell line, with IC_50_ values of 34.8, 38.1 and 33.6 μM for variabilin, Var-SLN and Ceramide, respectively. None of the samples revealed activity (>100 μM) against the colon (HT-29) and normal breast (MCF-12) cell lines. Var-SLNs induced 47% (vs. 38% for variabilin), 48 (vs. 29% for variabilin) and 59% (vs. 29% for variabilin) apoptosis in HT-29, MCF-7 and PC-3 cells, respectively. Furthermore, the “protective effect” provided by encapsulation into the SLNs on the non-tumorigenic epithelial cell line (MCF12A) is noteworthy. The incorporation of unstable or poorly soluble drugs into SLNs may provide a lifeline to compounds not previously considered as druggable due to poor physicochemical characteristics.

## Figures and Tables

**Table 1:** Dynamic Light Scattering (DLS) hydrodynamic radii and Zeta potential measurements for the SLNs.

	Hydrodynamic Radii (nm)			PDI	
Sample	t = 0	t = 14 days	Zeta Potential (mV)	t = 0	t = 14 days
Var-SLNs	198.2 ± 42.56	338.4 ± 57.70	−31.4 ± 5.16	0.129	0.136
Blank-SLNs	322.8 ± 36.02	824.1 ± 59.64	−18.3 ± 4.68	0.161	0.336

**Table 2:** IC_50_ (µM) of Var-SLN, Variabilin and Ceramide.

Cell Line	Var-SLN	Variabilin	Ceramide
PC-3	8.94	87.74	4.81
MCF-12	>100	>100	>100
MCF-7	34.83	38.08	33.61
HT-29	>100	>100	>100

## Supplementary Materials

The following are available online at https://www.mdpi.com/1420-3049/25/4/830/s1, Figure S1: Photograph of *Ircinia* sp. Sponge; Figure S2: ^1^H NMR spectrum of variabilin; Figure S3: ^13^C NMR spectrum of variabilin, and Figure S4: HR-ESIMS of variabilin.
