# Supplementary material for: Encapsulation of Variabilin in Stearic Acid Solid Lipid Nanoparticles Enhances Its Anticancer Activity in Vitro
Source: Molecules. 2020 Feb 14;25(4):830. doi: 10.3390/molecules25040830 (PMC7070932; doi:10.3390/molecules25040830)
Supplement: Supplementary file 1 [file molecules-25-00830-s001.pdf]

Supplementary material

Encapsulation of variabilin in stearic acid solid lipid nanoparticles enhances its anticancer activity *in vitro*

Mookho S. Lerata [1], Sarah D'Souza [1], Nicole R.S. Sibuyi [2], Admire Dube [1], Mervin E. Meyer [2], Toufiek Samaai [3], Edith M. Antunes [4] and Denzil R. Beukes [1,*]

Figure S1. Photograph of the sponge *Ircinia* sp. 1

Figure S2. ^{1}H NMR spectrum of mixture of12E- and 12Z-variabilin 2

Figure S3. ^{13}C NMR spectrum of mixture of12E- and 12Z-variabilin 2

Figure S4. HR-ESIMS spectrum of mixture of12E- and 12Z-variabilin 2

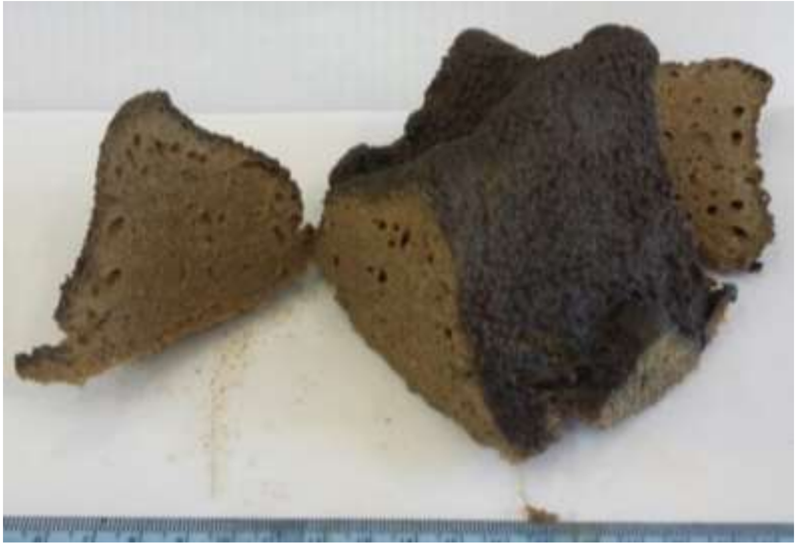

Figure S1. Photograph of the sponge *Ircinia* sp.

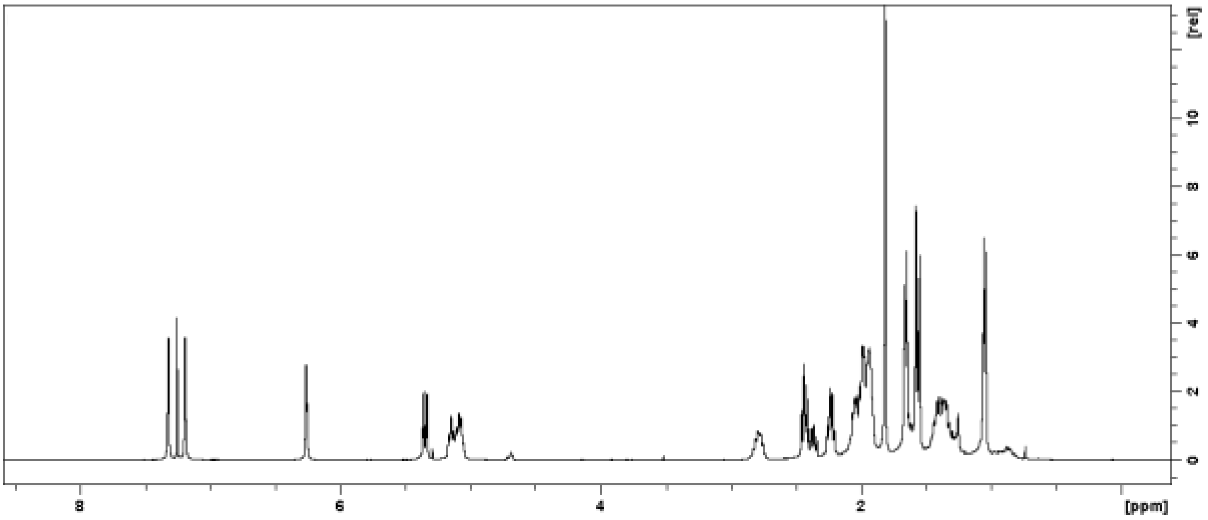

Figure S2. ^{1}H NMR spectrum of mixture of12*E*- and 12Z-variabilin

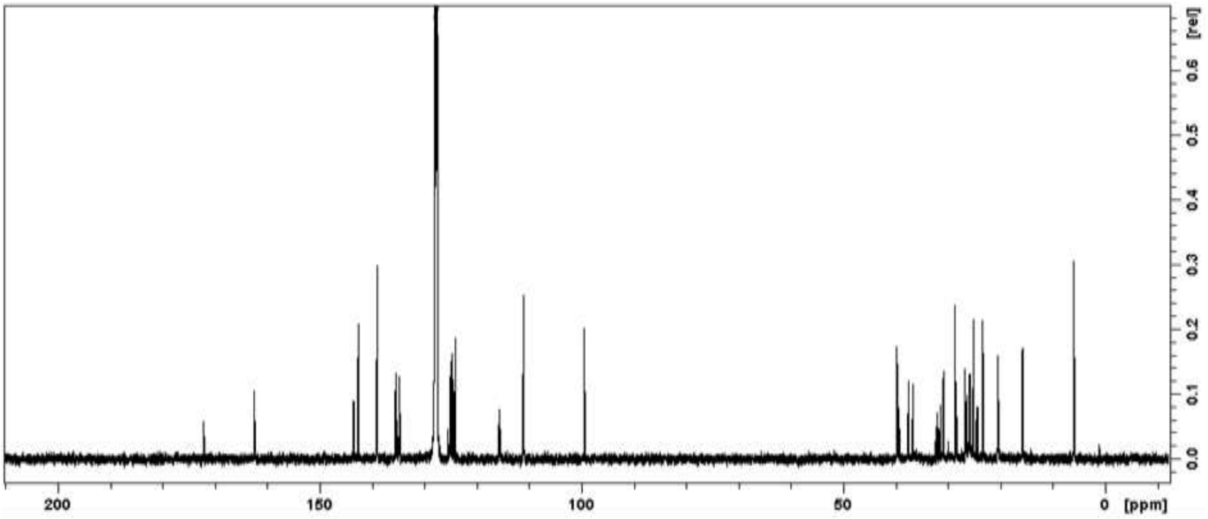

Figure S3. ^{13}C NMR spectrum of mixture of12E- and 12Z-variabilin

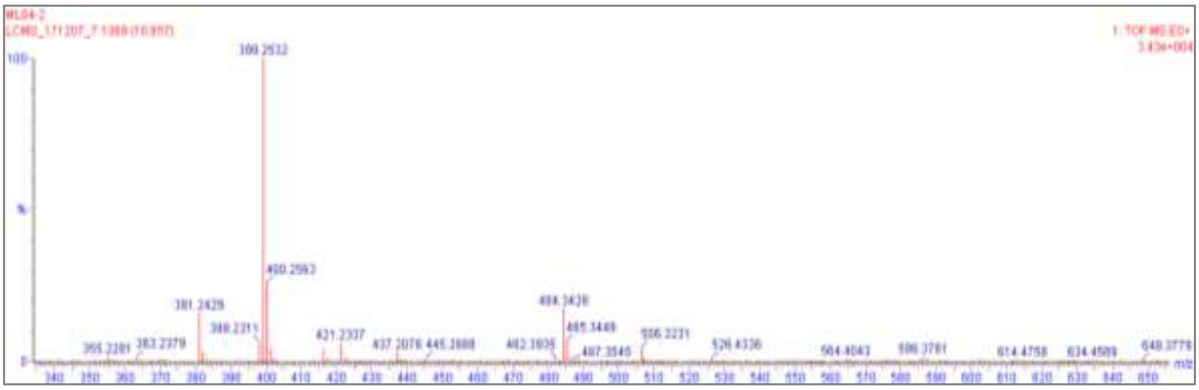

Figure S4. HR-ESIMS spectrum of mixture of12*E*- and 12Z-variabilin
